# Investigating the heavy metal (Hg, Cd, Pb, Cr, As) binding affinity and sensing capability of 2-((2-(hydroxymethyl)quinolin-8-yl)oxy)-N-(quinolin-8-yl)acetamide

**DOI:** 10.3389/fchem.2025.1665073

**Published:** 2025-09-08

**Authors:** Qianru Li, Shuting Jing, Liping He, Xianghe Kong, Wenbo Lan, Xiaofeng Wang

**Affiliations:** 1 School of Public Health, Xiangnan University, Chenzhou, Hunan, China; 2 Food Business Section, Municipal Center for Food and Drug Control, Chenzhou, Hunan, China; 3 School of Chemistry and Chemical Engineering, University of South China, Hengyang, Hunan, China

**Keywords:** “five toxic” heavy metal, density functional theory, 2-((2-(hydroxymethyl)quinolin-8-yl)oxy)-N-(quinolin-8-yl)acetamide, molecular simulation, solvent systems

## Abstract

Addressing severe environmental and health threats posed by mercury, cadmium, lead, chromium, and arsenic (“five toxic” heavy metals), this study employs density functional theory (DFT) calculations and molecular simulations to investigate the capture and detection mechanisms of the dual-fluorescent probe 2-((2-(hydroxymethyl)quinolin-8-yl)oxy)-N-(quinolin-8-yl)acetamide in both water solvent and dimethyl sulfoxide (DMSO). Key findings indicate that the probe forms highly stable, planar complexes with arsenic, lead, and chromium, exhibiting significant red-shifts in UV absorption bands and enhanced fluorescence intensity–strongest for arsenic in water solvent, while arsenic/chromium complexes show markedly increased fluorescence in DMSO. This work demonstrates the probe’s selective recognition of As, Pb, and Cr, with solvent polarity modulating detection signals, providing a novel theoretical framework for monitoring and remediating heavy metal pollution.

## 1 Introduction

Heavy metals exhibit non-biodegradability, persistence, and toxicity as environmental pollutants (Sirgedaitė-Šėžienė et al., 2025; Solgi et al., 2012). Specifically, mercury (Hg), cadmium (Cd), lead (Pb), chromium (Cr), and arsenic (As), collectively termed the “five toxic” heavy metals, induce harm to humans even at low doses through occupational exposure or food chains (Minnikova et al., 2017; Tchounwou et al., 2012). Hg is uniquely characterized by efficient biogeochemical cycling in ecosystems (Meng et al., 2010), damages central nervous systems, and induces toxic effects on oral mucosa, teeth, kidneys, and the cardiovascular system, while demonstrating genotoxicity and reproductive toxicity (Gentès et al., 2015). Moreover, prolonged exposure to high-concentration mercury may cause brain damage and even death (Ho et al., 2013). Cd exhibits immunotoxicity and reproductive toxicity, primarily accumulating in the kidneys (Ferraro et al., 2010; Hyder et al., 2013). It induces oxidative damage through multiple pathways: disrupting mitochondria to impair cellular respiration, activating heme oxidase to generate superoxide radical accumulation, and triggering dysregulated apoptosis via diverse mechanisms (Cuypers et al., 2010; Saed et al., 2010; Shaikh et al., 2009). Even under low-concentration chronic exposure, Cd significantly elevates the incidence of cancers such as prostate and lung cancer (Choi and Han, 2015; Kang et al., 2013; Wallin et al., 2016). Pb primarily accumulates in kidneys, liver, and central nervous system. By inhibiting cellular activity (Galano et al., 2014), it alters protein expression, causing chronic renal failure, cardiovascular diseases, and neurobehavioral damage. Effects on children are severe, with strong links to intellectual disabilities and developmental defects (Chen et al., 2012; Koedrith et al., 2013). Cr exists in multiple valence states in nature, among which Cr(VI) is a potent carcinogen. It induces malignant cell transformation (Huang et al., 2017), triggers cancer-related signaling pathways (Ge et al., 2019; Speer et al., 2021), promotes DNA damage (Browning et al., 2016), autophagy (Browning and Wise, 2017), and chromosomal instability (Proctor et al., 2014). Furthermore, Cr(VI) causes epigenetic alterations leading to lung, prostate, and bladder cancers, and is implicated in reproductive toxicity (He et al., 2013; Pratheeshkumar et al., 2016). As, being a metalloid, exhibits metabolic, neurotoxic, and developmental toxicity: it disrupts gut microbiota and impairs metabolic function (Hughes et al., 2011); inhibits intracellular enzyme activity, affecting glucose absorption, fatty acid β-oxidation, gluconeogenesis, and acetyl-CoA production (Olivares et al., 2016); and generates reactive oxygen/nitrogen species (RONS) (Valko et al., 2005). Moreover, As exposure induces methylation alterations in genomes, hepatocytes, and specific gene regulatory sequences (Koedrith et al., 2013; L et al., 2014).

Given the severe harm caused by the “five toxic” heavy metals to human health, establishing fast and efficient heavy-metal detection methods holds critical importance for environmental monitoring, food safety, and biomedical applications. Current commonly used heavy-metal-ion detection technologies include: Atomic Absorption Spectroscopy (AAS), X-ray Fluorescence Spectroscopy (XRF), High-Performance Liquid Chromatography (HPLC), Inductively Coupled Plasma Mass Spectrometry (ICP-MS), and Liquid Chromatography-Mass Spectrometry (LC-MS), etc. These analytical techniques generally deliver high sensitivity and accuracy in most scenarios; however, they involve costly instrumentation and inherent analytical constraints. AAS is influenced by sample pretreatment requirements, operational environment conditions, and instrument performance (Bacon et al., 2021). XRF accuracy suffers from significant matrix effects, necessitating specialized calibration protocols (Stosnach, 2005). HPLC exhibits insufficient sensitivity for trace heavy metal detection (Okano et al., 2015). ICP-MS incurs substantial maintenance costs (Mittal et al., 2017), while LC-MS demands advanced operator expertise and complex method development (Han et al., 2025). Fluorescence probe detection technology detects heavy metals by monitoring changes in fluorescence signals generated through specific binding between fluorescent probes and target metals. This method integrates molecular recognition with fluorescence phenomena, offering advantages such as operational simplicity, facile probe preparation, minimal instrumentation requirements, low detection limits, high sensitivity, cost-effectiveness, uniform signal distribution, and excellent reproducibility.

A fluorescent probe is generally a light-emitting molecule consisting of three units: a receptor (substrate), a fluorophore (signal unit), and a linker (Patil and Salunke-Gawali, 2018). The receptor binds to the substrate, while the fluorophore serves as the signal source. When the substrate interacts with heavy metal ions, the fluorophore’s luminescent properties exhibit alterations, causing shifts in fluorescence intensity or wavelength (Rathod et al., 2020). These changes enable indirect quantification of metal ion concentrations. Commonly used fluorophores include rhodamines, quinolines, fluoresceins, polycyclic aromatic hydrocarbons, coumarins, naphthalimides, cyanine dyes, fluoroboradipyrroles, and thiazines (Bao et al., 2023; Kwon et al., 2021; Ma et al., 2021; Nolan and Lippard, 2008; Sang et al., 2023; Yang et al., 2022; Zhao et al., 2020). Quinoline itself has structural rigidity, a large conjugated system, and good water solubility, making it easy to form complexes with metal ions. Quinoline and its derivatives are excellent chelating agents for metal ions. Quinoline rings contain N heteroatoms with lone electron pairs, facilitating coordination with metal cations. This interaction modifies the Highest Occupied Molecular Orbital (HOMO) distribution, shifting ground-state HOMO from nitrogen atoms to π orbitals. Consequently, π-π* transitions are triggered, altering the complex’s fluorescence properties (Zhao et al., 2024). Further functionalization with diverse substituents at various positions endows quinoline-based fluorescent probes with unique photophysical characteristics (Kathirvel et al., 2024; Suguna et al., 2024), enhancing their suitability for metal ion detection.

**FIGURE 1:**
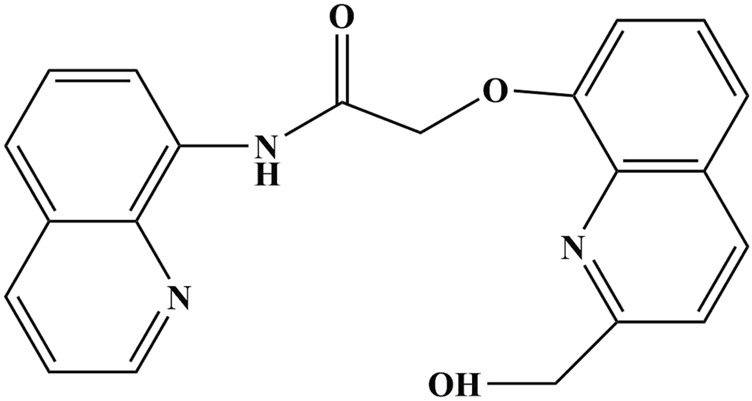
The molecular structure diagram of 2-((2-(hydroxymethyl)quinolin-8-yl)oxy)-N-(quinolin-8-yl)acetamide.

2-((2-(Hydroxymethyl)quinolin-8-yl)oxy)-N-(quinolin-8-yl)acetamide (Probe) is a quinoline-based fluorescent probe, exhibits good water solubility. As shown in Figure 1, the two quinoline rings are connected via amide bonds (-C(O)NH-) and an ether bond (-O-CH_2_-), making it a dual-emission fluorescent probe with an extended conjugated system. The planarity of the amide bonds and the moderate flexibility of the ether bond collectively enhance fluorescence resonance energy transfer efficiency while reducing signal attenuation caused by molecular vibration. Under specific conditions, its fluorescence intensity exceeds that of single-fluorophore probes. Previous studies have shown that the detection limit of this probe is 2.363 × 10^−8^ mol per liter (Lu et al., 2016), and the fluorescence intensity after binding with Cd is almost unaffected by anions, demonstrating excellent selectivity and a highly sensitive fluorescence enhancement response (Shi et al., 2015). Meanwhile, comparing aqueous phase interactions across different solvent systems offers critical insights for field detection of heavy metals. Fluorescence properties vary across solvents due to properties like polarity, refractive index, dielectric constant, and viscosity. Common solvent systems in practice include water, dimethyl sulfoxide (DMSO), acetonitrile, ethanol, and phosphate buffer (Shaily et al., 2018; Shi et al., 2015; Velmurugan et al., 2015; Wang et al., 2018). Water excels with intrinsic advantages: safety, environmental compatibility, and cost-effectiveness, while DMSO is favored for its solubility, thermal stability, and permeability. Studies on this probe’s binding with the “five toxic” heavy metals in water and DMSO systems hold critical importance for environmental detection.

With the rapid development of computer technology, the application of density functional theory (DFT) and the B3LYP method in computational simulation has been demonstrated to show a high degree of consistency with experimental results (Chen et al., 2017; Degem et al., 2022). Previous benchmark studies have confirmed the reliability of LanL2MB for heavy metal systems such as mercury, lead, and cadmium, especially when combined with hybrid functionals (Lan et al., 2025). In this study, the B3LYP method within DFT was used to investigate the capture of heavy metals such as mercury, cadmium, lead, chromium, and arsenic by dual fluorescent probes in the environment. If significant probe detection signals are observed or strong capture ability for certain heavy metal ions in specific solvents is found, it will provide important data support for the detection, recovery, and treatment of heavy metals in the environment.

## 2 Materials and methods

In this study, all calculations were performed using the Gaussian 16 B.01 quantum computational chemistry software based on DFT at the B3LYP level. The 6-311G* basis set applied to C, O, H, and N atoms, while LanL2MB served for the “five toxic” heavy metal ions (Hg, Cd, Pb, Cr, As). Geometric optimizations of Probe-metal complexes utilized the SMD solvation model in water and dimethyl sulfoxide (DMSO). All optimized geometries underwent vibrational frequency analysis at the same level of theory, confirming the absence of imaginary frequencies (true energy minima). Based on these validated structures, the structural parameters, Wiberg bond indices (WBIs), and infrared vibrational spectra of the complexes were systematically investigated. Binding energies were recalculated with Grimme’s dispersion correction (B3LYP-D3) to account for non-covalent interactions. Frontier molecular orbital analyses (HOMO/LUMO energies and energy gaps) were subsequently performed. Furthermore, the ultraviolet-visible absorption spectra were calculated via TD-DFT for the optimized molecular structures in both water and DMSO solvent environments, followed by the calculation of the fluorescence spectra based on S_1_-optimized geometries.

## 3 Research results and analysis

### 3.1 Molecular structure optimization

**FIGURE 2:**
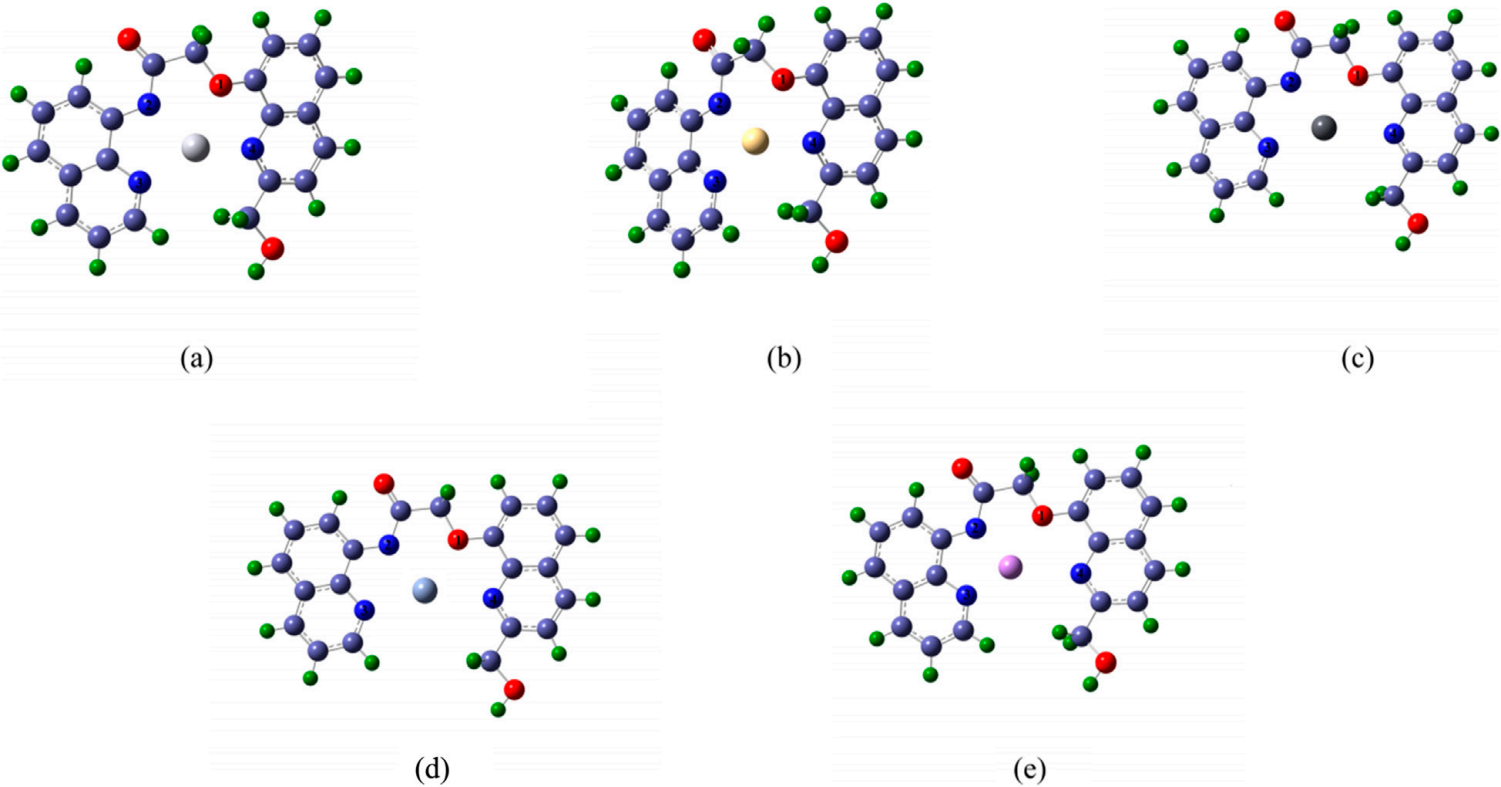
The schematic diagram of molecular structure optimization after the Probe forms complexes with Hg, Cd, Pb, Cr and As **(a)** complex-Hg, **(b)** complex-Cd, **(c)** complex-Pb, **(d)** complex- Cr, **(e)** complex-As.

The complexes formed by the double fluorescent Probe with Hg, Cd, Pb, Cr, and As were simulated, and their structures were optimized. Figure 2 shows the optimized structures of these complexes, in which the red atom represents oxygen (Atom 1), and the dark blue atoms represent nitrogen (Atoms 2, 3, and 4 in counterclockwise order). Light blue atoms denote carbon, green atoms hydrogen, while centrally positioned colored atoms represent the “five toxic” heavy metals, optimized geometries of Probe–metal complexes illustrating coordination cavities, with Hg (orange), Cd (teal), Pb (gray), Cr (blue), and As (purple).

Figure 2 further reveals that each heavy metal ion occupies the Probe’s cavity. Structural variations arise in the cavity upon metal binding due to differences in coordination geometry and atomic radius. The Probe’s extended conjugated system exhibits potential for selective metal ion recognition, enabling distinct characteristic signals.

### 3.2 Structural parameters

Table 1 summarizes bond lengths, bond angles, and dihedral angles from structural optimizations of the “five toxic” heavy metal complexes in Figure 2. These data fundamentally characterize coordination between the probe cavity’s N/O atoms and the central metal ion, along with the coordination cavity configuration.

**TABLE 1:** Bond length (Å), bond angle (°) and dihedral angle (°) of complexes.

Complex	Complex-Hg	Complex-Cd	Complex-Pb	Complex-Cr	Complex-As
O1-X	2.499	2.425	2.503	2.536	2.396
N2-X	2.359	2.241	2.263	2.305	2.045
N3-X	2.523	2.400	2.416	2.421	2.094
N4-X	2.583	2.400	2.842	2.771	2.845
O1-X-N2	66.437	71.269	70.524	69.461	74.761
N2-X-N3	70.377	73.736	70.377	72.094	79.994
N3-X-N4	113.549	105.132	156.193	157.489	143.323
N4-X-O1	64.240	69.681	60.678	60.755	61.913
O1-X-N2-N3	151.738	164.364	−179.489	−179.117	179.371
N2-X-N3-N4	108.875	100.592	175.401	170.322	−176.064
N3-X-N4-O1	123.242	140.141	177.386	173.427	−178.785
N4-X-O1-N2	135.345	113.559	177.432	175.517	−176.846

From the bond lengths between each heavy metal atom and the O1, N2, N3, and N4 atoms, data show that heavy metal atoms are not centered within the complexes, consistent with Figure 2 observations. Among the five complexes, N4-X bond lengths are generally greater than those of O1-X, N2-X, and N3-X—except for the Cd complex. Notably, even for smaller-radius atoms like As, the N4-X distance exceeds typical bonding ranges (e.g., N4-As). This suggests that when binding smaller metals, the primary binding force originates from O1, N2, and N3 atoms, potentially reducing probe-metal binding energy. As shown in Table 1, the metal is positioned closer to N2, resulting in the highest N2-X bond energy.

Among the “five toxic” heavy metal complexes, the N3-X-N4 bond angle exceeds others, enhancing coordination rigidity and reinforcing complex structural stability. This rigidity effectively restricts rotational/distortional motions. Notably, Pb, Cr, and As complexes exhibit dihedral angles O1-X-N2-N3, N2-X-N3-N4, N3-X-N4-O1, and N4-X-O1-N2 all approaching 180°, indicating near-coplanar alignment of metal atoms (Pb, Cr, As) with coordinating atoms. This planarity satisfies key fluorescence conditions: conjugated system, rigid structure, and planar geometry (Patrick et al., 2012; Zhang et al., 2005), enabling selective detection of Pb, Cr, and As ions.

Generally, longer bond lengths between identical atoms correspond to lower bond energies and weaker interatomic forces, while shorter bonds exhibit higher energies. Atoms with larger radii form significantly longer bonds with identical coordinating atoms than smaller-radius atoms. Given their position in the sixth period, Hg and Pb have substantially larger atomic radii than the other studied metals. Cd (fifth period) exhibits an intermediate radius, whereas As and Cr (fourth period) possess the smallest radii. Analysis of dihedral angles (Table 1) confirms that the Hg atom deviates from the plane of the probe molecule. Comprehensive analysis reveals that arsenic exhibits significantly shorter bond lengths than chromium, which likely contributes to the probe’s substantially stronger binding affinity for As. Although lead and mercury possess comparable atomic radii, the Hg complex deviates markedly from the rigid planar configuration, whereas the Pb complex maintains near-planar geometry—this structural distinction confers superior binding strength to lead relative to Hg. Concurrently, pronounced structural distortion in the cadmium complex likely underlies its weakest binding affinity among the five toxic heavy metals.

### 3.3 Wiberg bond indices (WBIs)

WBIs quantify bond strength and stability, reflecting bond multiplicity (e.g., single, double, or triple bonds). Higher bond orders generally indicate stronger bonds, higher bond dissociation energies, and enhanced stability (Dong et al., 2021). Table 2 shows the WBIs of the complexes formed by the double fluorescent Probe and the “five toxic” heavy metals. As evidenced in Table 2, while the bond energies of N2-X vary across complexes formed with different heavy metal ions, the WBIs of N2-X consistently ranks highest among all coordination bonds. This indicates greater electron density in bonding orbitals for N_2_-X interactions, correlating with enhanced bond strength. Notably, the N2-As bond exhibits the highest WBIs within the N2-X series, while N3-As similarly dominates the N3-X group—a pattern suggesting that arsenic likely achieves superior binding affinity with the probe through these optimized orbital interactions. This correlation with the shortest N2-X bond length (Table 1) effectively restricts molecular stretching vibrations, inhibiting vibrational and rotational motions, thus enhancing molecular rigidity. Notably, in complex-Pb, complex-Cr, and complex-As, the probe ligands and central atoms form a nearly planar structure, as presented in Table 1. This coplanarity facilitates distinct molecular fluorescence in complex-Pb, complex-Cr, and complex-As.

**TABLE 2:** Comparison of WBIs bond sequences of complexes.

Complex	Complex-Hg	Complex-Cd	Complex-Pb	Complex-Cr	Complex-As
O1-X	0.209	0.234	0.231	0.206	0.206
N2-X	0.510	0.549	0.680	0.610	0.768
N3-X	0.337	0.375	0.418	0.415	0.659
N4-X	0.328	0.396	0.174	0.189	0.094

### 3.4 Infrared vibration spectrum

**FIGURE 3:**
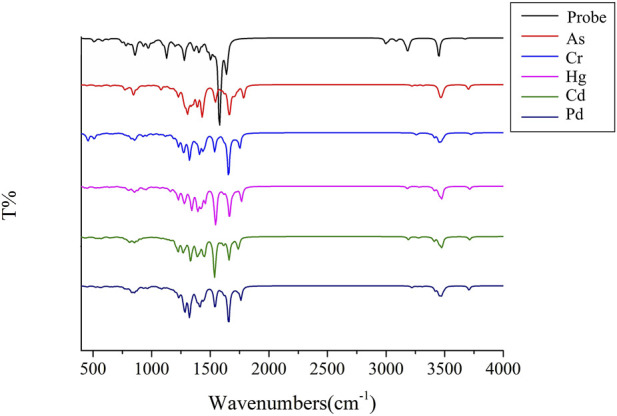
The infrared vibration spectra of Probe and complexes.

Figure 3 presents the simulated infrared vibration spectrum of the Probe complexes with the “five toxic” heavy metal ions (Hg, Cd, Pb, Cr, As) after structural optimization.

As shown in Figure 3, upon binding to heavy metal ions, the Probe’s original vibration peaks at 3,186 cm^−1^ and 3,453 cm^−1^ vanish. Both the probe and its complexes exhibit fingerprint peaks with absorption in the positive wavenumber range and no virtual frequencies, indicating that the optimized complex structure is stable. This confirms that the preceding structural parameters and Wiberg bond indices (WBIs) correspond to fully optimized geometries, providing a rigorous foundation for subsequent ultraviolet-visible (UV) absorption and fluorescence intensity analyses across varied solvent systems.

### 3.5 UV absorption spectrum

**FIGURE 4:**
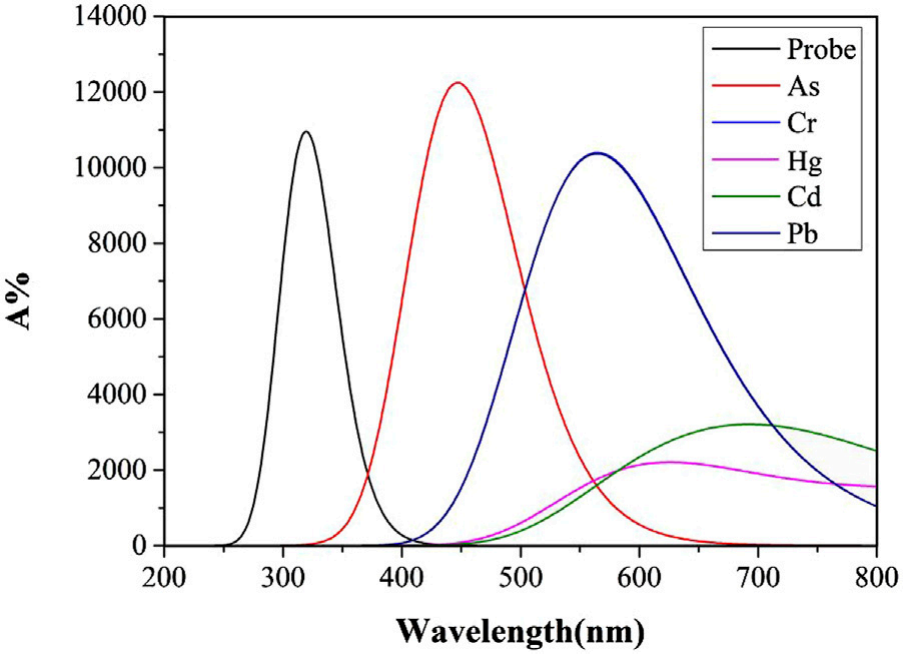
The Ultraviolet-visible absorption spectra of Probe and complexes in water solvent.

**FIGURE 5:**
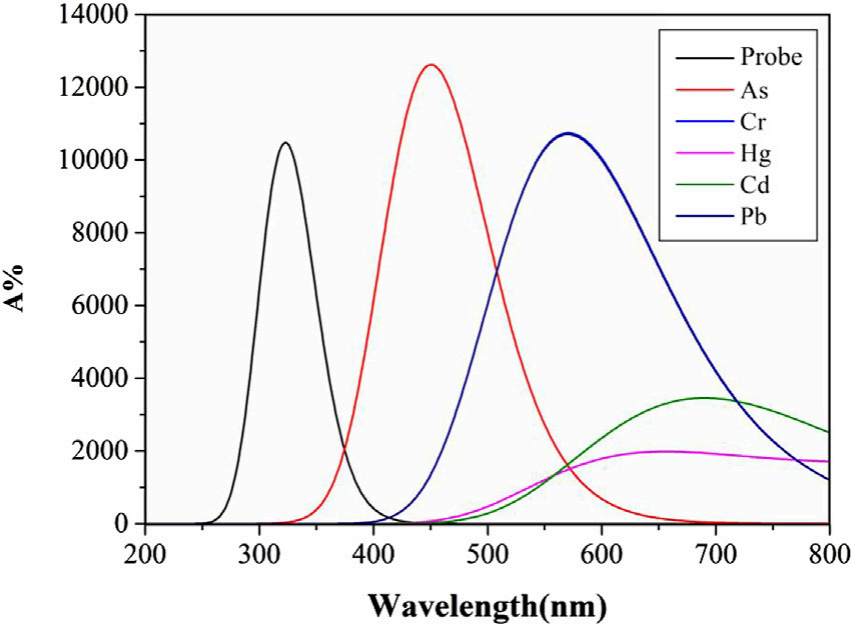
The Ultraviolet-visible absorption spectra of Probe and complexes in DMSO solvent.

Molecular structural alterations or complex formation are often reflected in ultraviolet-visible absorption spectrum, manifested by shifts in absorption peaks. Highly stable complexes typically exhibit distinct electronic transition energy-level differences, resulting in absorption peak shifts toward shorter wavelengths. Solvent polarity and temperature influence molecular energy levels and intermolecular interactions, thereby modulating ultraviolet-visible absorption characteristics (Tervola et al., 2020). Figures 4, 5 display UV absorption spectra of the probe and its “five toxic” heavy metal complexes in water and DMSO solvents. As shown in Table 3, the maximum absorption wavelengths are 309 nm (probe), 447 nm (As), 565 nm (Cr), 626 nm (Hg), 692 nm (Cd), and 565 nm (Pb) in water, and 331 nm (probe), 451 nm (As), 571 nm (Cr), 656 nm (Hg), 699 nm (Cd), and 571 nm (Pb) in DMSO. Critically, Cr and Pb complexes exhibit peak overlap (565 nm/water; 571 nm/DMSO), impeding spectral discrimination without significant solvent-dependent variation.

**TABLE 3:** The maximum UV absorption wavelengths (nm) of probe and complexes in two common solvent systems.

Solvent	Probe	Complex-As	Complex-Cr	Complex-Hg	Complex-Cd	Complex-Pb
Water	320	442	566	626	692	565
DMSO	322	451	571	656	690	571

Notably, the probe and its complexes exhibit nearly identical UV absorption profiles in Figures 4, 5, with λmax remaining virtually unchanged. This indicates that solvent polarity effects are negligible during UV spectroscopic detection of heavy metals using this probe. Critically, As binding induces a red-shift in the probe’s absorption peak with enhanced intensity compared to the free Probe, providing theoretical guidance for developing novel As quantification methods. However, Pb and Cd complexes remain spectrally indistinguishable via UV spectroscopy. Although complexation moderately reduces absorption for other metals, their distinct peaks enable clear differentiation, offering detection benchmarks for these ions.

### 3.6 Fluorescence spectrum

**FIGURE 6:**
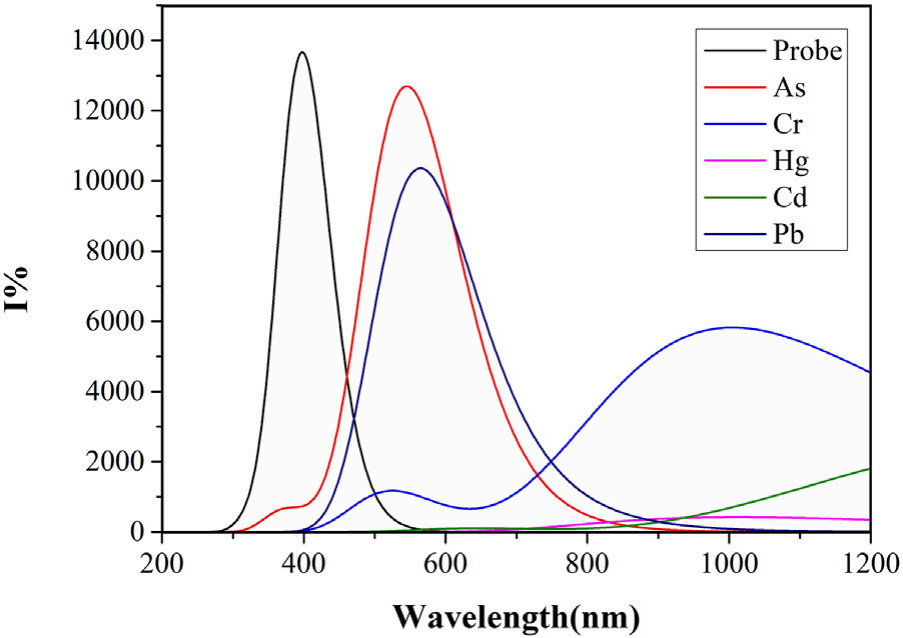
The fluorescence spectra of Probe and complexes in water solvent.

**FIGURE 7:**
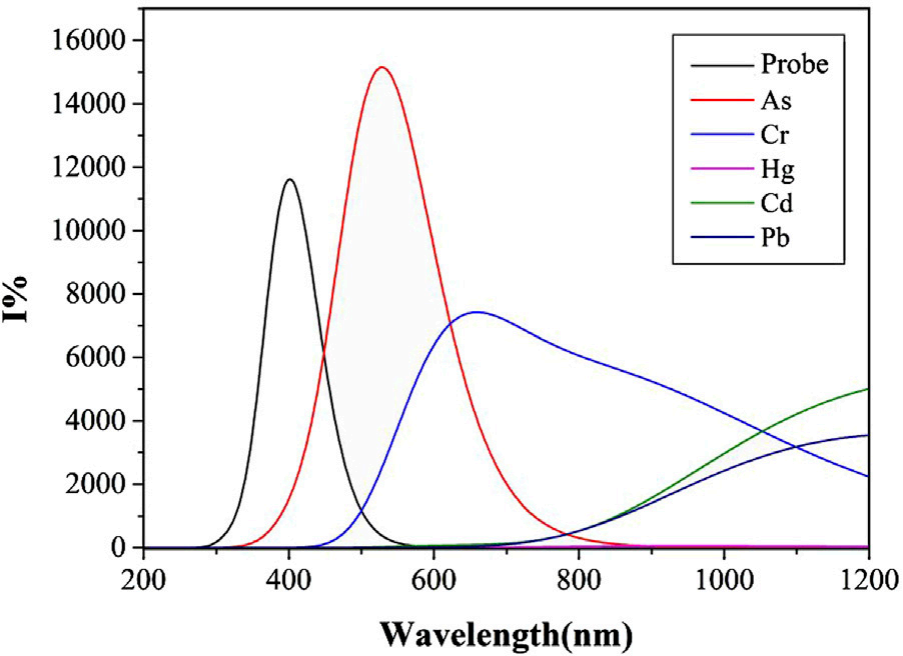
Fluorescence spectra of Probe and complexes in DMSO solvent.

Quinoline itself has a large conjugated system. Generally speaking, the larger the conjugated system, the higher the fluorescence efficiency is (Wang et al., 2009). However, for some metal ions, coordination may destroy the planar conjugated structure of quinoline due to an inappropriate cavity, thus affecting the fluorescence intensity. Different solvents may also affect the fluorescence intensity due to their different properties. In this study, the calculation of the fluorescence spectra based on S_1_-optimized geometries, and the fluorescence spectra of the Probe and its complexes with “five toxci” heavy metal ions were simulated in two commonly used solvents, water and DMSO. Figure 6 displays fluorescence spectra in water solvent, while Figure 7 shows those in DMSO solvent. Due to differences in ionic coordination modes and ionic radii, the coordination properties of the Probe vary significantly after binding to heavy metal ions.

As shown in Figure 6, the water solvent system exhibits significant variations in fluorescence intensity among the complexes formed by heavy metal ions and the Probe. complex-As demonstrates the highest fluorescence intensity with an optimal emission wavelength at 544 nm, followed by complex-Pb with a maximum emission wavelength of 564 nm. Binding with the Probe induces a distinct red-shift for both As and Pb complexes. In contrast, complex-Cd and complex-Hg show negligible fluorescence emission, potentially due to compromised conjugated systems or molecular planarity. Notably, arsenic forms the shortest bond length with N_2_, while lead exhibits the longest bond length with N_4_. The shorter As-N_2_ bond length suggests stronger bond energy and higher complex stability, consistent with its enhanced fluorescence intensity.

Unlike the simulation results of similar ultraviolet-visible absorption spectra under different solvents, the fluorescence spectra simulations using water and DMSO as solvent systems show marked differences. As shown in Figure 7, complex-As exhibits the strongest fluorescence, with a maximum emission wavelength of 529 nm; complex-Cr follows, with its maximum emission wavelength at 659 nm. When compared to the water solvent system, the fluorescence intensities of complex-As and complex-Cr were significantly enhanced. Notably, the maximum emission peak of complex-Cr showed a significant blue-shift, with its maximum emission wavelength decreasing from 1,010 to 659 nm. Notably, the fluorescence intensity of complex-Pb decreased significantly, indicating that for Pb determination using this probe, stronger fluorescence signals are detected in water or polar solvents. For As detection through molecular fluorescence spectrophotometry, distinctly different signals emerge in both water and organic solvent environments. Within organic solvents, the fluorescence signal originating from the complex-As exceeds that of the unbound probe itself.

### 3.7 Binding energy

Binding energy refers to the energy needed to break the coordination bond between the central ion and the ligand. The calculation method of binding energy is shown in Equation 1, ΔW_1_ represents the binding energy, W_1_ represents the total energy of the interacting system, W_2_ and W_3_ represents the two independent molecules energy of Probe and the “five toxic” heavy meals, respectively. It is a crucial parameter that can effectively reflect the stability of a substance. Generally, greater binding energy correlates with higher stability of the complex.

$$\Delta W_{1}=W_{1}-W_{2}-W_{3}$$ (1)

Table 4 presents the binding energies of complexes formed between the Probe and the “five toxic” heavy metal ions. The data demonstrate high thermodynamic stability across all systems, with the arsenic complex exhibiting exceptional binding strength (−20,593.37 kJ/mol)—consistent with metalloid-characteristic orbital hybridization. Notably, the lead complex ranks second (−12,010.61 kJ/mol), a phenomenon likely attributable to its near-perfect planar coordination geometry (Section 3.1; Figure 2C and Section 3.2; Table 1), which confers ∼10-fold greater affinity than the nonplanar mercury complex. This trend aligns precisely with our structural analysis in Section 3.1. Although the bond lengths of Cd differ little from those of Cr and As, Table 1 shows that the dihedral angles of their coordination bonds with probe ligands place Cd nearly in the same plane as Cr and As. When the probe forms a complex with cadmium ions, it induces severe distortion of the original planar configuration at the probe’s binding site, consequently diminishing the complex’s binding energy. By contrast, As and Pb complexes maintain superior planarity (as evidenced by dihedral angles approaching 180° in Section 3.2 and Table 1), which correlates with their enhanced binding energies. This indicates that the probe has potential for treating lead and mercury contamination in water, and its strong capture ability for Pb and Hg ions could lead to effective remediation outcomes.

**TABLE 4:** Binding energy (kJ/mol) of complexes.

Complex	Complex-Hg	Complex-Cd	Complex-Pb	Complex-Cr	Complex-As
Binding energy	−3,290.01	−161.34	−12010.61	−6,852.23	−20593.36

### 3.8 Frontier molecular orbitals (FMO)

**FIGURE 8:**
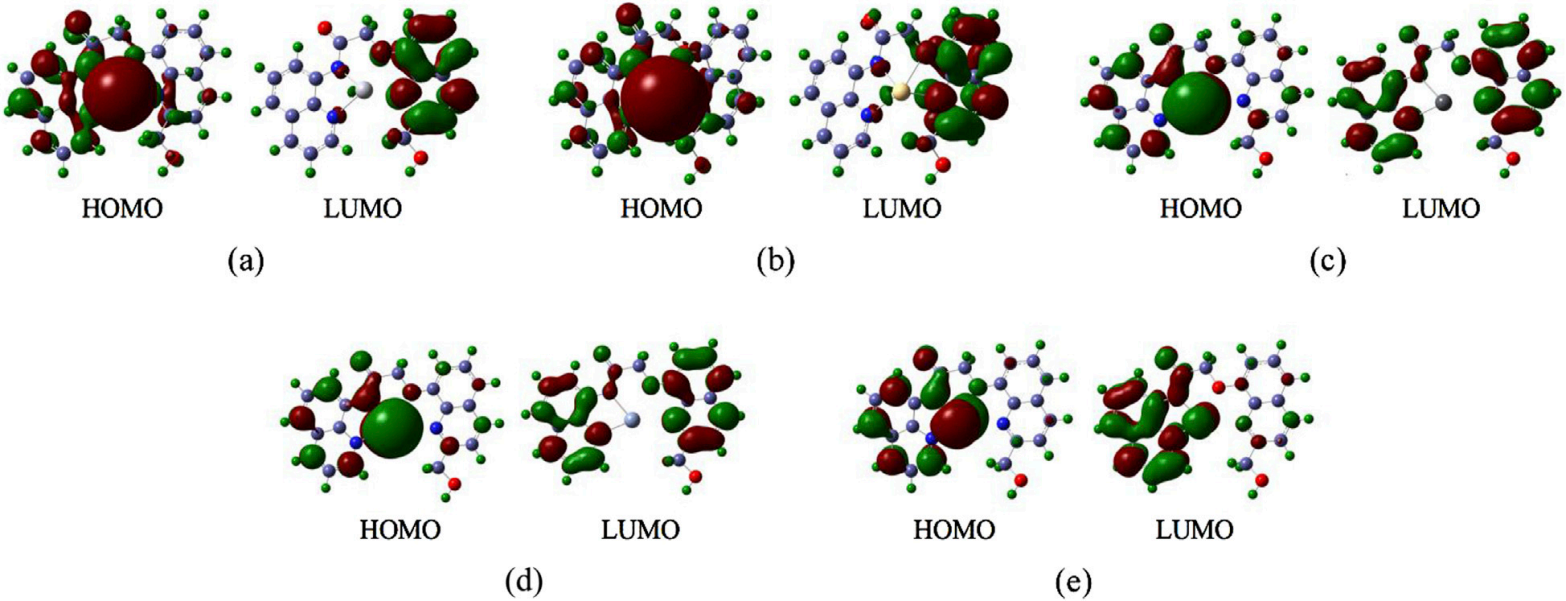
The Frontier Molecular Orbitals of the complexes **(a)** complex-Hg, **(b)** complex-Cd, **(c)** complex-Pb, **(d)** complex- Cr, **(e)** complex-As.

Generally, the HOMO (Highest Occupied Molecular Orbital) represents the orbital from which an electron is most easily removed, relating to the molecule’s ionization potential. The LUMO (Lowest Unoccupied Molecular Orbital) represents the orbital that most readily accepts an electron, relating to the molecule’s electron affinity (Baciu et al., 2024; Wei et al., 2020). The energy gap is the difference in energy between the HOMO and the LUMO. A large energy gap signifies that: A significant amount of energy (high excitation energy) is required to promote an electron from the HOMO to the LUMO. And The molecule is highly stable in its ground-state electronic configuration and exhibits low chemical reactivity (kinetic stability). The FMO energy levels—specifically the Highest Occupied Molecular Orbital (HOMO) and Lowest Unoccupied Molecular Orbital (LUMO)—for each complex are illustrated in Figure 8. Subfigures (a), (b), (c), (d), and (e) correspond to the complex-Hg, complex-Cd, complex-Pb, complex-Cr, and complex-As, respectively. The respective HOMO/LUMO energies (E_HOMO_/E_LUMO_) and their energy gaps (Eg) (in eV) are quantitatively summarized in Table 5.

**TABLE 5:** The energy of the HOMO/LUMO and Energy Gaps for the Complexes.

Complex	Complex-Hg	Complex-Cd	Complex-Pb	Complex-Cr	Complex-As
E_HOMO_	−2.5927	−2.1987	−1.9938	−1.9952	−2.4142
E_LUMO_	−1.0182	−0.4964	−0.3565	−0.3584	0.3317
Eg	−1.5745	−1.7023	−1.6373	−1.6368	−2.7459

Frontier molecular orbital (FMO) analysis reveals critical electronic structure determinants governing probe-metal interactions. The HOMO represents the ionization-susceptible electron-donating orbital, while the LUMO corresponds to the electron-accepting orbital, with their energy difference (ΔEg dictating excitation requirements and kinetic stability. As quantified in Table 5 and visualized in Figure 8, the arsenic complex exhibits the largest ΔEg (−2.7459 eV)—consistent with its shortened bond lengths (Section 3.1) and elevated Wiberg bond orders (Section 3.2). The cadmium complex demonstrates the second-largest ΔEg (−1.7023 eV) due to high excitation barriers arising from its symmetric coordination geometry, though HOMO localization on the cadmium center (Figure 8b) explains its diminished binding affinity. Critically, arsenic’s uniquely large Eg coupled with its exceptional binding energy (−20,593 kJ/mol, Table 4) confirms the probe’s selective recognition and thermodynamic stabilization of arsenic species, establishing a dual electronic-structural basis for preferential detection.

## 4 Conclusion

This study demonstrates that the dual-fluorescent probe 2-((2-(hydroxymethyl)quinolin-8-yl)oxy)-N-(quinolin-8-yl)acetamide achieves selective capture and detection of arsenic (As), lead (Pb), and chromium (Cr) among the “five toxic” heavy metals. Theoretical simulations reveal that the formed As/Pb/Cr complexes exhibit high structural stability and distinct spectroscopic responses: significant UV-Vis absorption red-shifts and enhanced fluorescence intensity. Critically, solvent polarity modulates detection performance—aqueous environments optimize Pb recognition while dimethyl sulfoxide (DMSO) amplifies fluorescence signals for As and Cr by 2–3-fold. The probe’s strong binding affinity for Pb and As further suggests utility in water remediation. These findings establish a versatile platform for environmental heavy metal monitoring and pollution control strategies.

## Data Availability

The original contributions presented in the study are included in the article/supplementary material, further inquiries can be directed to the corresponding authors.

## References

[B1] Baciu B. C. Bronk P. J. Guijarro A. (2024). Design and synthesis of thiahelicenes for molecular electronics. Front. Chem. 12, 1471413. 10.3389/fchem.2024.1471413 39469418 PMC11513318

[B2] Bacon J. R. Butler O. T. Cairns W. R. Cavoura O. Cook J. M. Davidson C. M. (2021). Atomic spectrometry update - a review of advances in environmental analysis. J. Anal. Atomic Spectrom. 36, 10–55. 10.1039/D0JA90074E

[B3] Bao X. Cao X. Ai K. Cui Y. Han Z. Zhou B. (2023). A dual-reaction-site and dual-channel Fluorescent-On probe for selectively monitoring mitochondrial glutathione. Sens. Actuators B Chem. 382, 133563. 10.1016/j.snb.2023.133563

[B4] Browning C. L. Wise J. P. Sr (2017). Prolonged exposure to particulate chromate inhibits RAD51 nuclear import mediator proteins. Toxicol. Appl. Pharmacol. 331, 101–107. 10.1016/j.taap.2017.05.030 28554658 PMC5568470

[B5] Browning C. L. Qin Q. Kelly D. F. Prakash R. Vanoli F. Jasin M. (2016). Prolonged particulate hexavalent chromium exposure suppresses homologous recombination repair in human lung cells. Toxicol. Sci. 153, 70–78. 10.1093/toxsci/kfw103 27449664 PMC5601504

[B6] Chen J. F. Chen Y. Liu W. Bai C. L. Liu X. X. Liu K. (2012). Developmental lead acetate exposure induces embryonic toxicity and memory deficit in adult zebrafish. Neurotoxicol Teratol. 34, 581–586. 10.1016/j.ntt.2012.09.001 22975620

[B7] Chen Y. Shi L. Guo S. Yuan Q. Chen X. Zhou J. (2017). A general strategy towards carbon nanosheets from triblock polymers as high-rate anode materials for lithium and sodium ion batteries. J. Mater. Chem. A 5, 19866–19874. 10.1039/C7TA06453E

[B8] Choi W. J. Han S. H. (2015). Blood cadmium is associated with osteoporosis in Obese males but not in non-obese males: the Korea national health and nutrition examination survey 2008–2011. Int. J. Environ. Res. Public Health 12, 12144–12157. 10.3390/ijerph121012144 26426028 PMC4626960

[B9] Cuypers A. Plusquin M. Remans T. Jozefczak M. Keunen E. Gielen H. (2010). Cadmium stress: an oxidative challenge. BioMetals 23, 927–940. 10.1007/s10534-010-9329-x 20361350

[B10] Degem N. Tamer O. Şimşek M. Avcı D. Yaman M. M. Başoğlu A. (2022). Experimental and theoretical approaches on structural, spectroscopic (FT-IR and UV-Vis), nonlinear optical, and molecular docking analyses for Zn(II) and Cu(II) complexes of 6-chloropyridine-2-carboxylic acid. Appl. Organomet. Chem. 36, e6678. 10.1002/aoc.6678

[B11] Dong D. Moriyoshi Y. Zhu J. (2021). To improve the performance of a variable geometry turbocharged SI engine by porous material application. Appl. Therm. Eng. 197, 117373. 10.1016/j.applthermaleng.2021.117373

[B12] Ferraro P. M. Costanzi S. Naticchia A. Sturniolo A. Gambaro G. (2010). Low level exposure to cadmium increases the risk of chronic kidney disease: analysis of the NHANES 1999–2006. BMC Public Health 10, 304–308. 10.1186/1471-2458-10-304 20525263 PMC2887827

[B13] Galano E. Arciello A. Piccoli R. Monti D. M. Amoresano A. (2014). A proteomic approach to investigate the effects of cadmium and lead on human primary renal cells. Metallomics 6, 587–597. 10.1039/c3mt00344b 24419708

[B14] Ge H. Li Z. Jiang L. Li Q. Geng C. Yao X. (2019). Cr (VI) induces crosstalk between apoptosis and autophagy through endoplasmic reticulum stress in A549 cells. Chem. Biol. Interact. 298, 35–42. 10.1016/j.cbi.2018.10.024 30416085

[B15] Gentès S. Maury-Brachet R. Feng C. Pedrero Z. Tessier E. Legeay A. (2015). Specific effects of dietary methylmercury and inorganic mercury in zebrafish (Danio rerio) determined by genetic, histological, and metallothionein responses. Environ. Sci. Technol. 49, 14560–14569. 10.1021/acs.est.5b03586 26509634

[B16] Han Y. Tian Y. Li Q. Yao T. Yao J. Zhang Z. (2025). Advances in detection technologies for pesticide residues and heavy metals in rice: a comprehensive review of spectroscopy, chromatography, and biosensors. Foods 14 (6), 1070. 10.3390/foods14061070 40232082 PMC11941943

[B17] He J. Qian X. Carpenter R. Xu Q. Wang L. Qi Y. (2013). Repression of miR-143 mediates Cr (VI)–induced tumor angiogenesis via IGF-IR/IRS1/ERK/IL-8 pathway. Toxicol. Sci. 134, 26–38. 10.1093/toxsci/kft101 23748240 PMC3693131

[B18] Ho N. Y. Yang L. Legradi J. Armant O. Takamiya M. Rastegar S. (2013). Gene responses in the central nervous system of zebrafish embryos exposed to the neurotoxicant methyl mercury. Environ. Sci. Technol. 47, 3316–3325. 10.1021/es3050967 23458150

[B19] Huang J. Wu G. Zeng R. Wang J. Cai R. Ho C. M. (2017). Chromium contributes to human bronchial epithelial cell carcinogenesis by activating Gli2 and inhibiting autophagy. Toxicol. Res. 6, 324–332. 10.1039/c6tx00372a 30090501 PMC6060711

[B20] Hughes M. F. Beck B. D. Chen Y. Lewis A. S. Thomas D. J. (2011). Arsenic exposure and toxicology: a historical perspective. Toxicol. Sci. 123, 305–332. 10.1093/toxsci/kfr184 21750349 PMC3179678

[B21] Hyder O. Chung M. Cosgrove D. Joseph M. H. Li Z. P. Amin F. (2013). Cadmium exposure and liver disease among US adults. J. Gastrointest. Surg. 17, 1265–1273. 10.1007/s11605-013-2210-9 23636881 PMC3974907

[B22] Kang M. Y. Cho S. H. Lim Y. H. Seo J. C. Hong Y. C. (2013). Effects of environmental cadmium exposure on liver function in adults. Occup. Environ. Med. 70, 268–273. 10.1136/oemed-2012-101063 23322921

[B23] Kathirvel R. Poongodi M. Vetriarasu V. Vivekanandan K. E. Selvakumar K. Obaid S. A. (2024). Quinoline-quinoline schiff-base as an effective chromogenic, fluorogenic, and smartphone assisted RGB detection of Pb^2+^ ion in near aqueous medium. Environ. Res. 250, 118530. 10.1016/j.envres.2024.118530 38387491

[B24] Koedrith P. Kim H. L. Weon J. I. Seo Y. R. (2013). Toxicogenomic approaches for understanding molecular mechanisms of heavy metal mutagenicity and carcinogenicity. Int. J. Hyg. Environ. Health 216, 587–598. 10.1016/j.ijheh.2013.02.010 23540489

[B25] Kwon N. Lim C. S. Ko G. Ha J. Lee D. Yin J. (2021). Fluorescence probe for imaging N-Methyl-D-Aspartate receptors and monitoring GSH selectively using two-photon microscopy. Anal. Chem. 93, 11612–11616. 10.1021/acs.analchem.1c02350 34382767

[B26] Lu K. Abo R. P. Schlieper K. A. Graffam M. E. Levine S. Wishnok J. S. (2014). Arsenic exposure perturbs the gut microbiome and its metabolic profile in mice: an integrated metagenomics and metabolomics analysis. Environ. Health Perspect. 122, 284–291. 10.1289/ehp.1307429 24413286 PMC3948040

[B27] Lan W. Meng Y. Wang X. He L. Li Q. Kong X. (2025). Analysis of heavy metal ions (Pb, Hg, Cr, Cd, As) capture and detection based on quinoline probe binding data. Sci. Rep. 15 (1), 9934. 10.1038/s41598-025-94856-8 40121294 PMC11929857

[B28] Lu H. L. Wang W. K. Tan X. X. Luo X. F. Zhang M. L. Zhang M. (2016). A new quinoline-based fluorescent probe for Cd^2+^ and Hg^2+^ with an opposite response in a 100% aqueous environment and live cell imaging. Dalton Trans. 45, 8174–8181. 10.1039/c6dt00362a 27093893

[B29] Ma Y. Yu Y. Mu X. Yu C. Zhou Y. Chen J. (2021). Enzyme-induced multicolor colorimetric and electrochemiluminescence sensor with a smartphone for visual and selective detection of Hg^2+^ . J. Hazard. Mater. 415, 125538. 10.1016/j.jhazmat.2021.125538 33721776

[B30] Meng B. Feng X. B. Qiu G. L. Cai Y. Wang D. Y. Li P. (2010). Distribution patterns of inorganic mercury and methylmercury in tissues of rice (Oryza sativa L.) plants and possible bioaccumulation pathways. J. Agric. Food Chem. 58, 4951–4958. 10.1021/jf904557x 20369851

[B31] Minnikova T. V. Denisova T. V. Mandzhieva S. S. Kolesnikov S. I. Minkina T. M. Chaplygin V. A. (2017). Assessing the effect of heavy metals from the novocherkassk power station emissions on the biological activity of soils in the adjacent areas. J. Geochem Explor 174, 70–78. 10.1016/j.gexplo.2016.06.007

[B32] Mittal M. Kumar K. Anghore D. Rawal R. K. (2017). ICP-MS: analytical method for identification and detection of elemental impurities. Curr. Drug Discov. Technol. 14 (2), 106–120. 10.2174/1570163813666161221141402 28003007

[B33] Nolan E. M. Lippard S. J. (2008). Tools and tactics for the optical detection of mercuric ion. Chem. Rev. 108, 3443–3480. 10.1021/cr068000q 18652512

[B34] Okano G. Igarashi S. Yamamoto Y. Saito S. Takagai Y. Ohtomo T. (2015). HPLC-spectrophotometric detection of trace heavy metals via 'cascade' separation and concentration. Int. J. Environ. Anal. Chem. 95 (2), 135–144. 10.1080/03067319.2014.994619

[B35] Olivares C. I. Field J. A. Simonich M. Tanguay R. L. Sierra-Alvarez R. (2016). Arsenic (III, V), indium (III), and gallium (III) toxicity to zebrafish embryos using a high-throughput multi-endpoint *in vivo* developmental and behavioral assay. Chemosphere 148, 361–368. 10.1016/j.chemosphere.2016.01.050 26824274 PMC4754138

[B36] Patil A. Salunke-Gawali S. (2018). Overview of the chemosensor ligands used for selective detection of anions and metal ions (Zn^2+^, Cu^2+^, Ni^2+^, Co^2+^, Fe^2+^, Hg^2+^). Inorg. Chim. Acta. 482, 99–112. 10.1016/j.ica.2018.05.026

[B37] Patrick M. Florian B. Katja L. Guntram R. René P. (2012). Cooperative Al(Salen)-Pyridinium catalysts for the asymmetric synthesis of trans-Configured β-Lactones by [2+2]-Cyclocondensation of acylbromides and aldehydes: investigation of pyridinium substituent effects. Molecules 17, 7121–7150. 10.3390/molecules17067121 22692239 PMC6268914

[B38] Pratheeshkumar P. Son Y. O. Divya S. P. Turcios L. Roy R. V. Hitron J. A. (2016). Hexavalent chromium induces malignant transformation of human lung bronchial epithelial cells via ROS-dependent activation of miR-21-PDCD4 signaling. Oncotarget 7, 51193–51210. 10.18632/oncotarget.9967 27323401 PMC5239469

[B39] Proctor D. M. Suh M. Campleman S. L. Thompson C. M. (2014). Assessment of the mode of action for hexavalent chromium-induced lung cancer following inhalation exposures. Toxicology 325, 160–179. 10.1016/j.tox.2014.08.009 25174529

[B40] Rathod R. V. Bera S. Maity P. Mondal D. (2020). Mechanochemical synthesis of a fluorescein-based sensor for the selective detection and removal of Hg^2+^ ions in industrial effluents. ACS Omega 5, 4982–4990. 10.1021/acsomega.9b03885 32201784 PMC7081412

[B41] Saed Z. Gavanji S. Davodi S. (2010). A review of genetic and epigenetic mechanisms in heavy metal carcinogenesis: nickel and cadmium. Int. J. Sci. Res. Environ. Sci. 1, 202–216. 10.12983/ijsres-2013-p202-216

[B42] Sang M. Huang Y. Liu Z. Li G. Wang Y. Yuan Z. (2023). Peroxynitrite/lipid droplet sequence-activated dual-lock fluorescent probes enable precise intraoperative imaging of atherosclerotic plaques. ACS Sens. 8, 893–903. 10.1021/acssensors.2c02590 36757333

[B43] Shaikh Z. A. Vu T. T. Zaman K. (2009). Oxidative stress as a mechanism of chronic cadmium-induced hepatotoxicity and renal toxicity and protection by antioxidants. Toxicol. Appl. Pharmacol. 154, 256–263. 10.1006/taap.1998.8586 9931285

[B44] Shaily K. A. Parveen I. Ahmed N. (2018). Highly selective and sensitive Coumarin–Triazole‐Based fluorometric 'turn‐off' sensor for detection of Pb^2+^ ions. Luminescence. 33, 713–721. 10.1002/bio.3468 29498808

[B45] Shi Z. Han Q. Yang L. Yang H. Tang X. Dou W. (2015). A highly selective two-photon fluorescent probe for detection of Cadmium(II) based on intramolecular electron transfer and its imaging in living cells. Chemistry–A Eur. J. 21 (1), 290–297. 10.1002/chem.201404224 25346036

[B46] Sirgedaitė-Šėžienė V. Striganavičiūtė G. Šilanskienė M. Kniuipytė I. Praspaliauskas M. Vaškevičienė I. (2025). Evaluating Populus tremula L. and Salix caprea L. for phytoremediation: growth, metal uptake, and biochemical responses under arsenic, cadmium, and lead stress. Front. Plant Sci. 16, 1617432. 10.3389/fpls.2025.1617432 40822729 PMC12354478

[B47] Solgi E. Esmaili-sari A. Riyahi-bakhtiari A. Hadipour M. (2012). Soil contamination of metals in the three industrial estates, Arak, Iran. Bull. Environ. Contam. Toxicol. 88, 634–638. 10.1007/s00128-012-0553-7 22323051

[B48] Speer R. M. Toyoda J. H. Croom-Perez T. J. Liu K. J. Pierce W. J. (2021). Particulate hexavalent chromium inhibits E2F1 leading to reduced RAD51 nuclear foci formation in human lung cells. Toxicol. Sci. 181, 35–46. 10.1093/toxsci/kfab019 33677506 PMC8081024

[B49] Stosnach H. (2005). Environmental trace-element analysis using a benchtop total reflection X-ray fluorescence spectrometer. Anal. Sci. 21 (7), 873–876. 10.2116/analsci.21.873 16038513

[B50] Suguna S. Velmurugan K. Parimaladevi D. Abiram A. Sukitha P. M. Kannan V. R. (2024). Quinoline scaffolds as fluorescent symmetric dipodal molecular cleft for swift and efficient Ag^+^ ion detection: applications in real samples and bioimaging. J. Photochem. Photobiol. A Chem. 447, 115226. 10.1016/j.jphotochem.2023.115226

[B51] Tchounwou P. B. Yedjou C. G. Patlolla A. K. Sutton D. J. (2012). Heavy metal toxicity and the environment. Mol. Clin. Environ. Toxicol. 3, 133–164. 10.1007/978-3-7643-8340-4_6 22945569 PMC4144270

[B52] Tervola E. Truong K. E. Ward J. S. Priimagi A. Rissanen K. (2020). Fluorescence enhancement of quinolines by protonation. RSC Adv. 10, 29385–29393. 10.1039/D0RA04691D 35521145 PMC9055970

[B53] Valko M. Morris H. Cronin M. T. D. (2005). Metals, toxicity and oxidative stress. Curr. Med. Chem. 12, 1161–1208. 10.2174/0929867053764635 15892631

[B54] Velmurugan K. Raman A. Don D. Tang L. Easwaramoorthi S. Nandhakumar R. (2015). Quinoline benzimidazole-conjugate for the highly selective detection of Zn(II) by dual colorimetric and fluorescent Turn-On responses. RSC Adv. 5, 44463–44469. 10.1039/C5RA04523A

[B55] Wallin M. Barregard L. Sallsten G. Lundh T. Karlsson M. K. Lorentzon M. (2016). Low‐level cadmium exposure is associated with decreased bone mineral density and increased risk of incident fractures in elderly men: the MrOS Sweden study. J. Bone Min. Res. 31, 732–741. 10.1002/jbmr.2743 26572678 PMC4832374

[B56] Wang J. Yang Z. Y. Wang B. D. Yi X. Y. Liu Y. C. (2009). Synthesis, characterization and DNA-binding properties of Ln(III) complexes with 6-Ethoxy Chromone-3-Carbaldehyde benzoyl hydrazone. J. Fluoresc. 19, 847–856. 10.1007/s10895-009-0482-y 19370403

[B57] Wang P. Fu J. Yao K. Chang Y. Xu K. Xu Y. (2018). A novel quinoline-derived fluorescent “Turn-On” probe for Cu^2+^ with highly selectivity and sensitivity and its application in cell imaging. Sens. Actuators B Chem. 273, 1070–1076. 10.1016/j.snb.2018.07.028

[B58] Wei W. Ren W. Jian W. Xia B. Li W. Bai F. Q. (2020). Stability, aromaticity, and photophysical behaviors of macrocyclic molecules: a theoretical analysis. Front. Chem. 8, 776. 10.3389/fchem.2020.00776 33102432 PMC7500243

[B59] Yang X. Zhang D. Ye Y. Zhao Y. (2022). Recent advances in multifunctional fluorescent probes for viscosity and analytes. Coord. Chem. Rev. 453, 214336. 10.1016/j.ccr.2021.214336

[B60] Zhang H. Xiang S. Li C. (2005). Enantioselective epoxidation of unfunctionalised olefins catalyzed by Mn(salen) complexes immobilized in porous materials via phenyl sulfonic group. Chem. Commun. 36, 1209–1211. 10.1039/B417041E 15726194

[B61] Zhao X. Yuan G. Ding H. Zhou L. Lin Q. (2020). A TP-FRET-Based fluorescent sensor for ratiometric visualization of selenocysteine derivatives in living cells tissues and zebrafish. J. Hazard. Mater. 381, 120918. 10.1016/j.jhazmat.2019.120918 31421550

[B62] Zhao J. Y. Zhang G. Hao H. C. Sun R. Xu Y. J. Ge J. F. (2024). Fluorescent probes based on quinoline and naphthidine derivatives with NIR and AIE properties for real-time monitoring mitochondrial viscosity during mitophagy. Sens. Actuators B Chem. 401, 135010. 10.1016/j.snb.2023.135010

